# Fatty Acid Profile and Biological Activities of Linseed and Rapeseed Oils

**DOI:** 10.3390/molecules201219887

**Published:** 2015-12-21

**Authors:** Anna Lewinska, Jacek Zebrowski, Magdalena Duda, Anna Gorka, Maciej Wnuk

**Affiliations:** 1 Department of Biochemistry and Cell Biology, University of Rzeszow, Zelwerowicza 4, 35-601 Rzeszow, Poland; alewinska@o2.pl; 2 Department of Plant Physiology, University of Rzeszow, Werynia 502, 36-100 Kolbuszowa, Poland; jaze28@interia.pl; 3 Department of Botany, University of Rzeszow, Werynia 502, 36-100 Kolbuszowa, Poland; duda.ma@wp.pl; 4 Department of Animal Physiology and Reproduction, University of Rzeszow, Werynia 502, 36-100 Kolbuszowa, Poland; agorka@ur.edu.pl; 5 Department of Genetics, University of Rzeszow, Rejtana 16C, 35-959 Rzeszow, Poland

**Keywords:** linseed oil, rapeseed oil, FTIR spectroscopy, gas chromatography, scratch wound healing assay, fibroblasts

## Abstract

It has been postulated that fatty acids found in edible oils may exert beneficial health effects by the modulation of signaling pathways regulating cell differentiation and proliferation, especially in the treatment of cardiovascular diseases. In the present study, the biological effects of selected edible oils—linseed (LO) and rapeseed (RO) oils—were tested *in vitro* on fibroblast cells. The fatty acid profile of the oils was determined using gas chromatography and FTIR spectroscopy. LO was found to be rich in α-linolenic acid (ALA), whereas oleic acid was the most abundant species in RO. Fatty acids were taken up by the cells and promoted cell proliferation. No oxidative stress-mediated cytotoxic or genotoxic effects were observed after oil stimulation. Oils ameliorated the process of wound healing as judged by improved migration of fibroblasts to the wounding area. As ALA-rich LO exhibited the most potent wound healing activity, ALA may be considered a candidate for promoting the observed effect.

## 1. Introduction

Flax or linseed (*Linum usitatissimum*, the *Linaceae* family), as a source of oil and fiber, is a widely used crop plant in food and textile industries [1]. It has been speculated that the lipid components of flax, especially omega-3 fatty acids, may have beneficial health effects [1]. Nevertheless, it has been also suggested that more human trials are needed to confirm the protective role of flaxseed products against coronary artery disease or hyperlipidemia [1]. Rapeseed (*Brassica napus*, the *Brassicaceae* family) is another source of oil for both nonfood and food uses [2]. Initially, the use of rapeseed oil as a diet supplement was limited due to its high levels of toxic erucic acid [2]. However, rapeseed cultivars were improved through traditional plant breeding and the low-erucic acid cultivar named canola was introduced [2]. Nowadays, canola oil is the third most important vegetable oil by volume after palm and soybean oil [2]. Generally, canola oil is characterized by low level of (7%) of saturated fatty acids (SFAs); substantial amounts of monounsaturated fatty acids (MUFAs) and polyunsaturated fatty acids (PUFAs), including 61% oleic acid, 21% linoleic acid, and 11% α-linolenic acid (ALA); plant sterols (0.53%–0.97%); and tocopherols (700–1200 ppm), all of which are considered cardioprotective substances [2,3,4]. More recently, using the PubMed search engine, experimental, epidemiological and clinical studies on the protective effects of rapeseed oil were summarized and evaluated [5]. It has been concluded that rapeseed-mediated effects were documented in short-term studies on the biomarkers of risk factors for cardiovascular diseases [5]. The involvement of ALA has been also postulated [5], although, the authors suggested that rapeseed oil cannot be recommended as a suitable substitute for extra-virgin olive oil as part of a Mediterranean-style diet [5].

As PUFA may affect the production of inflammatory mediators such as eicosanoids and cytokines, and stimulate epithelial cell proliferation *in vitro* [6,7], it also seems worthwhile to elucidate fatty acid-mediated effects during wound healing process that is accompanied by immune response and altered cell proliferative potential. In the present study, the biological activities of three edible plant-derived oils, namely two linseed oils and rapeseed oil were investigated and the fatty acid profile-biological activity relationships were established.

## 2. Results and Discussion

First, the fatty acid profiles of three commercially available edible oils, namely two linseed oils (VIS NATURA, LO and DARY NATURY, LO_c_) and rapeseed oil (VIS NATURA, RO) were investigated (Table 1 and Table 2).

**Table 1:** Fatty acid (FA) composition of the three oils used: total levels of MUFA, PUFA and SFA [%] and the ratio of unsaturated fatty acids to saturated fatty acids and MUFA to PUFA.

Oils	MUFA [%]	PUFA [%]	SFA [%]	Unsaturated FA/Saturated FA	MUFA/PUFA
LO	15.8	74.8	9.4	9.64	0.21
LO_c_	20.3	68.5	11	8.07	0.30
RO	62.9	29.6	7.5	12.33	2.10

Fatty acids were analyzed using gas chromatography (GC). LO, linseed oil (VIS NATURA); LO_c_, linseed oil (DARY NATURY); RO, rapeseed oil (VIS NATURA); MUFA, monounsaturated fatty acids; PUFA, polyunsaturated fatty acids; SFA, saturated fatty acids.

**Table 2:** Fatty acid (FA) composition of the three oils used: quantitative analysis of identified fatty acids.

Common Name, C:D ^a^	Oils		
	LO	LO_c_	RO
Myristic Acid, 14:0	<0.1	0.1	ND ^b^
Palmitic Acid, 16:0	5.1	5.3	4.3
Stearic Acid, 18:0	4.3	5.1	2.0
Arachidic Acid, 20:0	<0.1	0.3	0.8
Behenic Acid, 22:0	ND ^b^	0.2	0.4
Oleic acid, 18:1, *n*-9	15.8	20.0	62.5
Linoleic Acid, 18:2, *n*-6	16.5	17.8	19.6
α-Linolenic Acid, 18:3, *n*-3	58.3	50.7	10.0
Erucic Acid, 22:1, *n*-9	ND ^b^	0.3	0.4

^a^ C:D, lipid numbers, where C is the number of carbon atoms in the fatty acid and D is the number of double bonds in the fatty acid. If applicable, *n*-*x* nomenclature is also provided. ^b^ ND, not detected. LO, linseed oil (VIS NATURA); LO_c_, linseed oil (DARY NATURY); RO, rapeseed oil (VIS NATURA).

**Figure 1:**
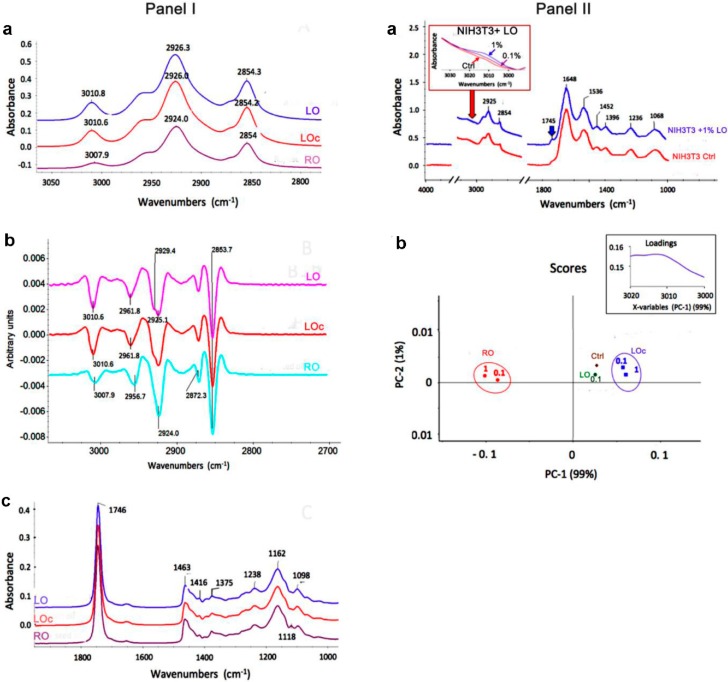
Chemical characteristics of oils using FTIR spectroscopy. **Panel I**: ATR-FTIR spectra of vegetable oils added to culture medium. (**a**) The spectra within the region between 3050 and 2800 cm^−1^ characteristic for alkene (above 3000 cm^−1^) and alkane (below 3000 cm^−1^) fatty acid chains; (**b**) The second derivatives of the oil spectra used for precise location of the absorbance peaks; (**c**) The spectra in the region of (C=O) stretching vibrations of ester group from lipids and in the fingerprint region (1500–1000 cm^−1^). **Panel II**: (**a**) ATR-FTIR spectra of LO-treated cells. Positions of major bands are shown. Bands related to fatty acid functional groups including olefinic (C-H) stretching absorbance in the region characteristic for unsaturated fatty acids and C=O stretching vibrations for esters characteristic for lipid compounds are denoted using red and blue arrows, respectively. The FTIR spectra in the region of =C-H bonds reflecting the presence of unsaturated acids within the cells are enlarged and shown within a red frame. The spectra were base-corrected and normalized to amide I peak at 1648 cm^−1^; (**b**) PCA two-dimensional score plot of FTIR spectra for principle components (PA) 1 and 2 in the region of unsaturated fatty acids (3200–3000 cm^−1^) derived from oil-treated cells. Oil-dependent and concentration-independent effects are observed. Corresponding loading values are shown in a black frame. LO, linseed oil (VIS NATURA); LO_c_, linseed oil (DARY NATURY); RO, rapeseed oil.

Gas chromatography (GC)-based analysis revealed that unsaturated fatty acids dominated over saturated fatty acids in all samples studied (Table 1). The high content of α-linolenic acid (ALA, omega-3 polyunsatured fatty acid) in both linseed oils used (LO and LO_c_) was observed (Table 2). In contrast, the level of ALA in the rapeseed oil (RO) was relatively low (Table 2). The most abundant unsaturated fatty acid in RO was oleic acid (omega-9 monounsaturated fatty acid) (Table 2). As expected, very low level of erucic acid was detected in low erucic acid RO used (Table 2). Oils were also characterized using FTIR spectroscopy (Figure 1).

Oils (0.1%) were added to cell culture medium used (DMEM) and infrared spectral features within the ranges specific for fatty acids and lipids were investigated (Figure 1, Panel I). The ATR-FTIR spectra in the absorbance region between 3050 and 2800 cm^−1^ that is relevant for typical olefinic and aliphatic molecular structures in these compounds are shown (Figure 1, Panel Ia). In general, similar spectral patterns with prominent absorbance bands at about 3010 cm^−1^ assigned to alkene (*cis* = C-H) stretching vibrations, at about 2925 cm^−1^ corresponding to asymmetric stretching of methylene (–CH_2_) group and at 2854 cm^−1^ reflecting symmetric stretches of the methylene group [8] were observed in all analyzed oils. The C-H stretching vibration bands for methyl (-CH_3_) group were found only as shoulders at about 2960 cm^−1^ and 2872 cm^−1^, precisely resolved after second derivative processing of the acquired data (Figure 1, Panel Ib). The unsaturated (*cis* = C-H) stretching band was of similar locations for both linseed oils and was shifted to higher energy compared to the rapeseed oil. The difference in the position of the peak in this band for LO, LO_c_ and RO indicates the difference in the degree of unsaturation and in the relative content of various fatty acids [9]. Indeed, linseed oil is particularly rich in ALA [10], whereas rapeseed oil contains mostly oleic and linoleic fatty acids [11] that is also in agreement with our GC-based data (Table 2). The shift toward higher frequency and higher absorbance intensity of the olefinic band for LO and LO_c_ relative to RO reflected both higher content and higher degree of unsaturation in linseed oils. All oils showed the highest absorbance band at 1746 cm^−1^ assigned to C=O stretching vibrations and no absorbance at about 1710 cm^−1^ characteristic for carboxylic C=O stretching vibrations that indicates the presence of fatty acids exclusively in ester forms [8] (Figure 1, Panel Ic). The oils also showed typically distinct bands at 1463 cm^−1^ and at 1375 cm^−1^ corresponding to scissoring CH_2_ and symmetric CH_3_ deformation modes, respectively. The esterification of fatty acids was confirmed by only weak absorbance at 1416 cm^−1^ (O-H banding vibrations), 1238 cm^−1^ (C-O stretching vibrations) and the occurrence of a relatively prominent band at 1162 cm^−1^ (C-O stretching bond) (Figure 1, Panel Ic). Moreover, we have analyzed infrared spectra of oil-treated cells (Figure 1, Panel IIa). The appearance of the absorbance bands at 3010 cm^−1^ (red arrow) and a high peak at 1743 cm^−1^ (blue arrow), specific for C=O stretching vibrations of ester group found in lipids, confirms the cellular uptake of unsaturated fatty acids in oil-treated cells (Figure 1, Panel IIa). The effect on the absorbance in the region of *cis* = C-H stretching mode reflecting the presence of unsaturated fatty acids was concentration-dependent (Figure 1, Panel IIa, enlarged spectrum in a red frame). The spectral features in the regions characteristic for proteins and nucleic acids were not affected (Figure 1, Panel IIa). The shape and the height of IR absorbance bands specific for proteins located at 1648 cm^−1^, 1536 cm^−1^, and 1452 cm^−1^, corresponding to amide I, II and III bands, respectively, were similar for untreated and oil-treated cells. The absorbance at 1236 cm^−1^ and 1068 cm^−1^ assigned to phosphate (PO_2_^−^) anti-symmetric and symmetric stretching modes of phosphodiester groups in nucleic acids, respectively, was also unchanged that is in agreement with previous observations [8,12]. The FTIR spectra of oil-treated cells were further resolved using chemometric approach and Principle Component Analysis (PCA) (Figure 1, Panel IIb). The PC1 component explained 99% of spectral variability among examined samples. The pattern of clustering the scores showed that the type of oil but not the oil concentration affected qualitatively the spectral profile of the cells. Moreover, RO-treated cells showed much more altered metabolic profile in this region compared to LO-treated cells (Figure 1, Panel IIb). Taken together, fatty acids found in LO, LO_c_ and RO were taken up by the NIH3T3 cells.

**Figure 2:**
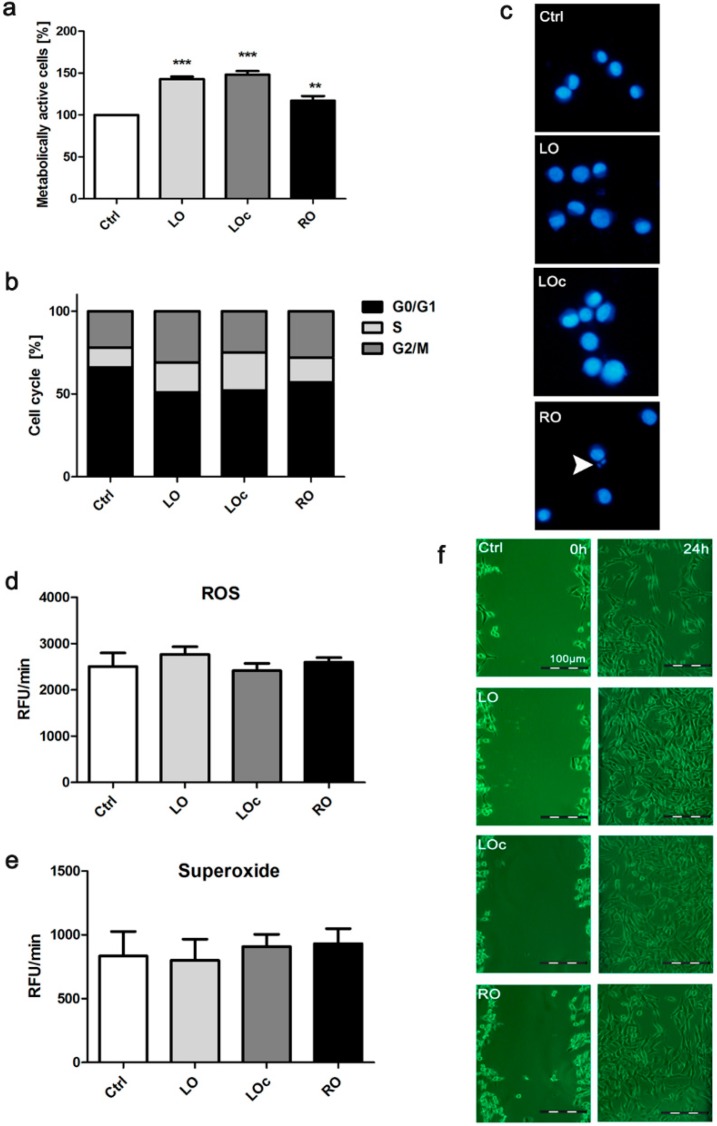
Oil-mediated effects on NIH3T3 cells. Fibroblasts were treated with 0.1% oils for 24 h. (**a**) Metabolic activity was assayed using MTT test; (**b**) DNA content-based cell cycle analysis; (**c**) Micronuclei formation. DNA was visualized using Hoechst 33342 staining (blue). Arrowhead indicates a micronucleus; (**d**) Reactive oxygen species (ROS) production. (**e**) Superoxide production; (**f**) Scratch wound healing assay. Cell migration was evaluated under an inverted microscope. Bars indicate SD, *n* = 3, ** *p* < 0.01, *** *p* < 0.001 compared to untreated control (ANOVA and Dunnett’s *a posteriori* test). LO, linseed oil (VIS NATURA); LO_c_, linseed oil (DARY NATURY); RO, rapeseed oil.

Second, we selected the concentration of 0.1% to study oil-mediated effects on fibroblasts. We found that all three oils enhanced metabolic activity of NIH3T3 cells (MTT assay) that may indicate that oils promoted cell proliferation (Figure 2a). LO-mediated stimulation was much more evident than RO-mediated stimulation. A 1.5- and 1.2-fold increase in the level of metabolically active cells was observed after LO and RO treatment, respectively (*p* < 0.001 and *p* < 0.01) (Figure 2a).

Oil-associated changes in the cell cycle were also observed and the effect of LO was the most accented (Figure 2b). Oils did not promote micronuclei formation, suggesting that the analyzed oils have no genotoxic potential (Figure 2c). However, a very low level of micronuclei (<1%) was observed after RO treatment (Figure 2c). Oils did not induce oxidative stress as judged by oil-mediated production of reactive oxygen species (ROS) and superoxide (Figure 2d,e). Taken together, oils at the concentration of 0.1% were not cytotoxic or genotoxic to NIH3T3 cells. In contrast, oils stimulated cell proliferation.

We also studied the ability of oils to promote wound healing *in vitro* (Figure 2f). A scratch wound healing assay was performed and we found that all three oils possessed wound healing activity compared to control conditions (Figure 2f). Oils promoted cell migration to the wounded area (Figure 2f). As the most spectacular effect was observed for LO treatment (Figure 2f) and LO is rich in ALA (Table 2), we speculate that ALA may facilitate wound healing *in vitro*.

It has been suggested the dietary polyunsaturated fatty acids (PUFA) may have potential health benefits, especially in the treatment of cardiovascular diseases, cancer, diabetes and other diseases by affecting immune response, cell-cell interactions, cell signaling, differentiation and proliferation [13,14,15]. PUFA-mediated modulation of cell proliferation and anti-inflammatory effects may also promote wound healing. There are contradictory results on PUFA-associated wound healing activity *in vitro* and *in vivo*, which may be explained at least in part by the different mode of administration (topical application *versus* diet supplementation), different concentrations, sources of PUFA and wound healing assays used [6,16,17,18]. It has been postulated and rebutted that PUFA may have stimulatory role in wound healing [6,16,17,18]. Thus, there is still a need for comprehensive evaluation of PUFA-mediated effects during wound healing process involving inflammation, cell proliferation and tissue remodeling [19]. Nitric oxide has been implicated in wound healing because inducible nitric oxide synthase (iNOS) deficiency caused significant impairment in wound healing [20]. The activation of the nuclear factor-kappaB (NF-κB) pathway and prostaglandin E_2_ (PGE_2_) may be linked by the cross-talk of iNOS and nitric oxide during collagen production and wound healing in PUFA-stimulated fibroblasts [18]. Eicosapentaenoic acid (EPA) treatment resulted in lower PGE_2_ levels and increased iNOS expression, nitric oxide production, collagen formation and recoverage area during wound healing compared to arachidonic acid (AA)-treated fibroblasts [18]. Moreover, lipopolysaccharide (LPS)-induced activation of NF-κB pathway resulted in more pronounced changes in the gene expression in EPA-treated fibroblasts compared to AA-treated cells [21]. The affected genes were toll-like receptor 4 (TLR4), adaptor proteins (TNF receptor-associated factor 6, TRAF6, myeloid differentiation primary response gene 88, MYD88), signal transduction kinases (NF-κB-inducing kinase, inhibitors of kappa light polypeptide gene enhancer isoforms), inhibitor protein (I-κB alpha chain), transcription factors (nuclear factor of kappa light chain gene enhancer, p105 and NF-κB subunit p100), DNA binding proteins (cAMP response element binding protein) and response genes known to affect collagen production (interleukin 6, IL-6, iNOS, monocyte chemotactic protein-1) [21]. Thus, it has been suggested that fatty acids may be used as adjuvants in combination with other therapies (e.g., selective targeting of the NF-κB pathway) to control collagen formation [21]. On the other hand, it should be also noted that a higher number of unsaturation may stimulate oxidation and cause a delay in wound healing. Indeed, inhibition of lipid peroxidation stimulated wound healing and angiogenesis, and restored impaired vascular endothelial growth factor (VEGF) expression in the genetically diabetic mouse [22]. Oleic acid rich diet also protected against oxidative modification of high density lipoprotein [23]. However, under our experimental conditions, the possibility that ALA-rich oil treatment may promote disequilibrium of cellular redox homeostasis and oxidative stress-mediated genotoxicity was ruled out as the production of ROS and superoxide and micronuclei generation were unaltered after LO stimulation.

## 3. Materials and Methods

### 3.1 Fatty Acid Profiles

Linseed and rapeseed oils (LO and RO) were purchased from VIS NATURA (Kamieniec Wroclawski, Poland, https://olejzycia.pl). Moreover, linseed oil (LOc) from DARY NATURY (Grodzisk, Poland, http://darynatury.pl) was used for comparison. The metabolite profiles of oils in the cell culture medium as well as after uptake by the cells were characterised using attenuated total reflectance Fourier transform infrared (ATR-FTIR) spectroscopy. Briefly, oil-treated cells were lyophilized and homogenized into powder using agate mortar and pestle. A drop of oil or a pinch of cell powder were deposited onto one-bounce diamond crystal of the ATR accessory (Smart Orbit, Thermo Scientific, Madison, WI, USA) coupled to iZ10 module of Nicolet iN10 MX microspectrometer (Thermo Fisher Scientific, Waltham, MA, USA) equipped with a deuterated triglycine sulfate (DTGS) detector and KBr beam splitter. Sixty four interferograms were collected and co-added within the wavelength range between 4000 and 400 cm^−1^ at the 4 cm^−1^ resolution. The ATR diamond crystal was cleaned carefully before successive measurements and possible presence of analyte residues on the crystal surfaces was examined by recording the residual spectra after cleaning. The spectra were averaged and preprocessed using advanced ATR-correction and rubber band baseline correction. Spectra acquired for oils were normalized to the absorbance at C=O (1746 cm^−1^) band, whereas these collected for the cells were normalized to the amide I peak. Band positions were precisely determined using Savitzky-Golay secondary derivative (set for 9 points and polynomial order of 3). Preprocessing and data analysis were performed by means of OMNIC (v. 8.1, Thermo Fisher Scientific) software. Principal component analysis was performed using Unscrambler X (v.10.1, CAMO, Oslo, Norway) software. Moreover, fatty acid composition of oils was analyzed using gas chromatography (GC). GC analysis was performed using CP-3800 gas chromatograph (Varian, Palo Alto, CA, USA) equipped with flame ionization detector and split/splitless injector. Injector temperature was set at 250 °C and samples were injected manually (1 µL) with split ratio of 1:30. Two different cyanopropyl silicone capillary columns were used: DB-225ms 30 m × 0.25 mm, with film thickness of 0.25 lm and DB-23 60 m × 0.25 mm, with film thickness of 0.25 lm. The temperature program was set from 60 °C to 220 °C at rate of 7 °C/min and was the same for both columns. Helium was used as a gas carrier at a flow rate of 1 mL/min (DB-225ms column) and of 1.5 mL/min (DB-23 column). Detector temperature was set at 280 °C. The following reference standard compounds were used: myristic acid (PHR1124, Sigma-Aldrich, St. Louis, MO, USA), palmitic acid (PHR1120, Sigma-Aldrich), stearic acid (PHR1114, Sigma-Aldrich), arachidic acid (39383, Sigma-Aldrich), behenic acid (11909, Sigma-Aldrich), oleic acid (75090, Sigma-Aldrich), linoleic acid (62230, Sigma-Aldrich), α-linolenic acid (62160, Sigma-Aldrich), erucic acid (45629, Sigma-Aldrich) and AOCS #3 Mix (10 components, 35024, Restek Corporation, Bellefonte, PA, USA). Star GC Workstation Version 6.4 chromatographic software (Varian) was used for data collection and calculation.

### 3.2 Cell Culture

Mouse embryo fibroblasts (NIH3T3, ATCC, Manassas, VA, USA) (3000 cells/cm^2^) were cultured at 37 °C in Dulbecco’s Modified Eagle’s medium (DMEM, Sigma-Aldrich) supplemented with 10% fetal calf serum (FCS) and an antibiotic and antimycotic mixed solution (100 U/mL penicillin, 0.1 mg/mL streptomycin and 0.25 μg/mL amphotericin B, Sigma-Aldrich) in a humidified atmosphere in the presence of 5% CO_2_ until they reached confluence. Typically, the cells were passaged by trypsinization and maintained in DMEM.

### 3.3 Cytotoxicity, Cell Cycle and Genotoxicity

Fibroblasts were treated for 24 h with 0.1% oils. Oils were dissolved in DMSO (Sigma-Aldrich) and added to the medium to a given final concentration. The DMSO concentration in the cell culture did not exceed 0.1% that did not influence the cell survival. Oil cytotoxic potential was then estimated using an MTT assay [24]. Cell cycle analysis was conducted as previously described [25]. Briefly, oil-treated cells were stained with a mixture of Hoechst 33342 (2.5 μg/mL) and CellTrace™ Calcein Red-Orange AM (2.5 μM) (Thermo Fisher Scientific) in a serum-free DMEM medium at 37 °C for 30 min, washed with PBS and subjected to cell cycle analysis using an In Cell Analyzer 2000 (GE Healthcare, Little Chalfont, UK) equipped with a high performance CCD camera. Micronuclei formation was checked after Hoechst 33342 (Thermo Fisher Scientific) staining [26].

### 3.4 Oxidative Stress

Fibroblasts were treated for 24 h with 0.1% oils. Intracellular reactive oxygen species (ROS) and superoxide production was then estimated using the fluorogenic probes 2′,7′-dichlorodihydrofluorescein diacetate (H_2_DCF-DA) and dihydroethidium, respectively [26].

### 3.5 Scratch Wound Healing Assay

An *in vitro* wound healing assay was used to evaluate the migration of NIH3T3 cells as described elsewhere [7]. Briefly, fibroblasts were cultured until approximately 90% confluency was reached and medium was then removed and fresh medium containing 0.1% oils was added. A sterile pipette tip was used to make a wound by streaking across a monolayer of NIH3T3 cells. The migration of cells into the wound was documented after 24 h post wounding using an inverted microscope.

### 3.6 Statistical Analysis

The results represent the mean ± SD from at least three independent experiments. Statistical significance was assessed by 1-way ANOVA using GraphPad Prism 5 (GraphPad Software, Inc., La Jolla, CA, USA), and with the Dunnett's multiple comparison test.

## 4. Conclusions

In summary, we have shown that LO may improve the process of wound healing *in vitro* and ALA may be a candidate responsible for observed effect.
